# Mimicking the Osteosarcoma Bone Microenvironment Using MEW‐Printed PCL Scaffolds

**DOI:** 10.1002/mabi.70224

**Published:** 2026-07-18

**Authors:** Chen Ye, Sabine Schulze, Richard Frank Richter, Max von Witzleben, Anne Weidlich, Hagen Fritzsche, Klaus‐Dieter Schaser

**Affiliations:** ^1^ University Center of Orthopaedic Trauma and Plastic Surgery University Hospital Carl Gustav Carus and Faculty of Medicine at TUD Dresden University of Technology Dresden Germany; ^2^ Center for Translational Bone Joint and Soft Tissue Research University Hospital Carl Gustav Carus and Faculty of Medicine at TUD Dresden University of Technology Dresden Germany

**Keywords:** bone microenvironment, MEW (melt electrowriting), osteosarcoma, PCL (polycaprolactone), SaOS‐2 cells

## Abstract

Osteosarcoma is the most common primary malignant bone tumor, typically affecting children and adolescents during rapid growth periods. With current treatments, the five‐year survival rate for metastatic or recurrent cases remains only 20–30%, highlighting the need for new therapeutic approaches and better preclinical models. Our goal is to simulate the osteosarcoma bone microenvironment using melt electrowriting (MEW)‐printed polycaprolactone (PCL) scaffolds with optimized calcium phosphate formation. To achieve this, we fabricated PCL scaffolds with distinct MEW micro‐architectures, including rectangular, triangular, and hexagonal designs with comparable primary fiber spacing, and investigated two calcium phosphate formation methods: natural cell‐generated mineralization and direct calcium phosphate cement coating. Using SaOS‐2 osteosarcoma cells, we systematically evaluated cell behavior, mineralization dynamics, and scaffold mechanical properties. This approach aims to establish a biomimetic 3D model that bridges the gap between laboratory findings and clinical applications, enabling more pathophysiologically relevant drug testing platforms.

## Graphical abstract

PCL scaffolds exhibiting different MEW‐derived microarchitectures were coated either through physiological cell‐mediated mineralization or via direct calcium phosphate cement (CPC) deposition. Their mechanical characteristics, together with the behavior of SaOS‐2 osteosarcoma cells cultured on the scaffolds, were investigated to determine their potential for mimicking the native bone microenvironment.

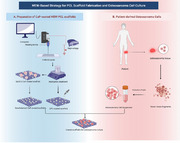

## 1 Introduction

OS (osteosarcoma) is the most prevalent primary malignant bone tumor and predominantly affects children and adolescents during periods of rapid skeletal growth [1]. It typically arises in the metaphysis of long bones, most frequently in the distal femur, proximal tibia, and proximal humerus [2]. Despite multimodal therapy combining surgery, polychemotherapy and, less frequently, radiation, the five‐year survival rate for metastatic or recurrent disease remains low (∼20–30%) [3, 4]. This highlights the need for improved therapeutic strategies and preclinical models that better capture drug resistance and help predict chemotherapy response [5].

A major obstacle is the complexity of the bone tumor microenvironment and the limited ability of current models to reproduce it [6, 7]. Bone‐specific mechanical properties, mineral composition, and multicellular interactions strongly influence osteosarcoma progression, metastasis, and drug tolerance [8, 9]. This unique environment, characterized by specific mechanical properties, mineral composition, and complex cellular interactions, significantly influences tumor behavior [10, 11]. The interplay between osteoblasts, osteoclasts, and osteosarcoma cells creates a dynamic environment that supports tumor growth and invasion [12].

Traditional 2D cell culture systems fail to capture these complex interactions, while animal models, although valuable, often do not fully represent human disease progression and treatment responses [13, 14]. Furthermore, the heterogeneity of osteosarcoma tumors and the influence of the surrounding stroma increase the difficulty of developing representative models [15]. These limitations contribute to the gap between preclinical findings and clinical outcomes, motivating more advanced in vitro systems [16].

Tissue engineering and bioprinting have enabled more complex 3D in vitro cancer models that better mimic native microenvironments [17, 18]. These approaches aim to recreate the 3D structure and the inorganic and organic composition in the bone tumor microenvironment [19]. Among additive manufacturing techniques, melt electrowriting (MEW) enables the fabrication of micro‐fiber scaffolds (down to ∼5 µm) with tunable dimensions and mechanical properties [20, 21]. Recent developments in additively manufactured scaffolds for regenerative bone‐mimetic applications highlight the value of combining architectural control with tailored material cues and mechanics‐informed evaluation, including surface‐modified printed polymers [22], hybrid polymer–hydrogel constructs [23], and multi‐morphology designs supported by imaging‐based modeling [24]. However, for osteosarcoma microenvironment modeling, it remains challenging to systematically isolate the effects of micro‐architecture, scaffold thickness, and mineralization route within one controllable platform while enabling clinically relevant tumor cell readouts.

Micro‐scale geometric control is particularly relevant because bone architecture provides structural cues that regulate cell behavior and tissue formation [25, 26]. MEW allows highly controlled fiber deposition at cell‐relevant length scales and the fabrication of architectures that mimic aspects of hierarchical bone organization [27], making it suitable for studying how structural cues modulate osteosarcoma cell behavior and drug responses [28]. Polycaprolactone (PCL) has become a preferred material for such applications due to its excellent processability, mechanical properties, and Food and Drug Administration (FDA) approval for certain medical applications [29, 30]. Its biocompatibility and slow degradation further support long‐term in vitro studies [31]. PCL‐based scaffolds have therefore been explored for bone tissue engineering and cancer modeling [32, 33], and combining PCL with MEW enables reproducible, biomimetic micro‐architectures [34].

Based on their potential to mimic various aspects of bone architecture and their influence on cell behavior [35], three different geometric designs—rectangular (R), triangular (T), and hexagonal (H)—were fabricated as PCL scaffolds using MEW technology [36]. For calcium phosphate (CaP) deposition, we tested two different methods of CaP formation in creating an artificial bone microenvironment for osteosarcoma modeling. One method is based on the natural deposition of CaP by SaOS‐2 (Sarcoma Osteogenic‐2) cells on the PCL scaffold, while the other involves direct coating of the scaffold with calcium phosphate cement (CPC). This study is novel in that it (i) quantified how architecture and layer number shape mineralization, mechanical performance, and cell distribution, (ii) provides a direct comparison of two mechanistically distinct mineralization routes (cell‐mediated CaP vs CPC coating), and (iii) incorporates patient‐derived osteosarcoma cells as a translational readout on mineralized scaffolds. We then relate mineralization and mechanical properties to tumor cell adhesion patterns to identify scaffold designs and coating routes that support in vitro osteosarcoma models.

## 2 Materials and Methods

### 2.1 Scaffold Fabrication

PCL scaffolds were fabricated using melt electrowriting (MEW) on a GeSiM BioScaffolder 3.1 equipped with a GeSiM MEW head (GeSiM mbH, Radeberg, Germany). Medical‐grade polycaprolactone (PCL, Purasorb PC12, Corbion, Amsterdam, Netherlands) pellets were heated and extruded through a 250 µm needle. Molten PCL fibers were deposited layer‐by‐layer onto the collector plate to create scaffolds with well‐defined architectures.

Pore size is defined as the center‐to‐center spacing between adjacent parallel fibers within a layer. For hexagonal architecture, two characteristic pore openings are present: the primary hexagonal opening (400 µm) and smaller secondary triangular openings (∼200 µm) intentionally incorporated within each unit. We report (i) the nominal designed pore openings for each architecture and (ii) the measured fiber spacing from microscopy (Table 1).

**TABLE 1:** Overview of the scaffolds used in this study (measurements refer to 40‐layer scaffolds unless stated otherwise). Projected fiber length density was used as a geometry‐derived descriptor of fiber content per projected scaffold area. For rectangular and triangular scaffolds, values were estimated from the sum of fiber families based on the nominal center‐to‐center spacing (∼400 µm). For the hexagonal scaffold, the value was approximated from the projected hexagon–triangle unit geometry and is therefore reported as a range (∼7.5–8.7 mm/mm2) rather than a direct measurement.

Name (abbreviation)	Fiber diameter [µm]	Fiber spacing (primary) [µm]	Projected fiber length density [mm/mm^2^]	Estimated thickness [µm]
Rectangular (R)	15.09 ± 0.67	397.1 ± 12.14	5.0	∼600 (40L × 15 µm)
Triangular (T)	15.26 ± 0.52	401.3 ± 13.99	7.5	∼600 (40L × 15 µm)
Hexagonal (H)	15.33 ± 0.77	402.3 ± 17.26 (primary; secondary 200 ± 14.78)	≈7.5–8.7	∼600 (40L × 15 µm)

The melt temperature was maintained at 79°C, air pressure at 400 kPa, and print head speed at 700 mm/min. A voltage of +7.5 kV was applied to the collector, while the nozzle was grounded. Printing was performed under ambient laboratory conditions. The average fiber diameter was 15.21 ± 0.71 µm.

Nominal scaffold thickness was not directly measured in this study. Instead, thickness was reported as a layer‐number–based nominal estimate calculated from the number of printed layers (N = 40 layers) and the measured single‐fiber diameter (Table 1). This estimate assumes ideal layer‐by‐layer stacking and therefore serves only as an approximation. In practice, material build‐up at fiber junctions and architecture‐dependent stacking/displacement can alter the local scaffold height, particularly at crossover points and with increasing layer counts. Accordingly, thickness values reported throughout the manuscript should be interpreted as nominal estimates, and layer number is used as the primary design parameter for comparing scaffold configurations.

Projected fiber length density was additionally used as a geometry‐derived descriptor of fiber content per projected scaffold area; its estimation is described in Table 1.

Scaffolds for cell experiments had a side length of 7 mm, while scaffolds for mechanical testing were 15 mm.

### 2.2 Calcium Phosphate Coating of Scaffolds

The scaffolds were treated with 1 m NaOH in 50% methanol to enhance the hydrophilicity of PCL. Following the treatment, the scaffolds were thoroughly rinsed with distilled water to remove any residual chemicals and then air‐dried. The scaffolds were sterilized under ultraviolet (UV) light to ensure aseptic conditions.

Two distinct approaches were employed to establish calcium phosphate (CaP) layers on the MEW‐printed PCL scaffolds:

#### 2.2.1 CPC Coating

MEW‐printed PCL scaffolds were coated with a clinically approved calcium phosphate cement (CPC; INNOTERE, Radebeul, Germany) by applying drops of a CPC/ethanol solution (0.01 g/ml (w/v), using 96% ethanol), followed by drying at room temperature for 48 h to form a uniform CPC layer.

#### 2.2.2 Cell‐Mediated Matrix Mineralization

SaOS‐2 cells are widely used in bone tissue engineering for scaffold coating with a native cell‐derived calcium phosphate (CaP) matrix, which mimics the natural bone mineralization process.

SaOS‐2 (ACC‐243, DSMZ Braunschweig, Germany) [37, 38] cells were expanded in McCoy's 5A medium (Bioconcept, Bioconcept AG, Switzerland) supplemented with 15% fetal calf serum (FCS; Corning Inc., NY, USA), 1% penicillin‐streptomycin (P/S; both Gibco, Life Technologies). Cultures were incubated at 37°C in a humidified atmosphere with 5% CO_2_. Media were replaced every 3 to 4 days, and cells were passaged when they reached approximately 80% confluency.

MEW‐printed scaffolds were placed in ultra‐low attachment (Corning, Corning, NY, USA) 12 and 24 multiwell plates containing cell culture medium for 15–30 min prior to cell seeding. Then, SaOS‐2 cells were added (1 × 10^4^ cells/cm^2^) by pipetting the cell suspension to the medium. Due to the surface treatment of the multiwell plates, cells primarily attached to the scaffolds. To induce osteogenic differentiation, the culture medium was changed to α‐MEM supplemented with GlutaMAX (Gibco, Thermo Fisher Scientific, Waltham, MA, USA), 10% heat‐inactivated Fetal Calf Serum (HI‐FCS; Biowest, Riverside, MO, USA), 1% P/S (100 U/mL penicillin and 100 µg/mL streptomycin (Gibco)), 10 mm β‐glycerophosphate, and 50 µm ascorbic acid (both from Sigma‐Aldrich, St. Louis, MO, USA). Medium was changed every 2–3 days. After 25 days, matrix deposition was stopped, the scaffolds were washed with sterile PBS (Phosphate Buffered Saline, ThermoFisher Scientific, USA) and decellularized by incubation with 20 mM ammonium hydroxide for 30 min. Scaffolds were washed by gently pipetting up and down, followed by three additional washing steps.

### 2.3 Cell Characterization

#### 2.3.1 Live/Dead Staining

Cell viability of SaOS‐2 seeded on the MEW‐printed PCL scaffolds was assessed using a Live/Dead staining assay at days 3, 11, and 25 of culture. Cells were labeled with calcein AM and EthD‐1 (ThermoFisher Scientific LIVE/DEAD Viability/Cytotoxicity Kit). Visualization was carried out by fluorescence microscopy (Keyence BZ 9000, Osaka, Japan).

#### 2.3.2 Image Analysis and Quantification

Live/dead images were analyzed using ImageJ (NIH, USA). For Live/Dead assays, green (calcein‐AM, live) and red (EthD‐1, dead) channels were separated. For each field of view, the visible scaffold region was manually selected as the region of interest (ROI). Areas outside the visible scaffold region were excluded from the analysis. Background was corrected using the same procedure for all images, and a fixed threshold was applied to each channel across all groups and time points. Because cell‐deposited CaP can show autofluorescence, the measured live‐cell and dead‐cell covered area fractions may include a minor contribution from CaP‐related background signal. Since this potential background could affect both channels, it is expected to have only a minor impact on the relative comparison between groups. Live‐cell covered area fraction and dead‐cell area fraction were calculated as the percentage of thresholded green or red signal area relative to this ROI. Viability (%) was calculated from area fractions as Live/(Live + Dead) × 100%. Three independent scaffolds were analyzed per group (n = 3), and three randomly selected fields of view per scaffold were quantified; values were averaged per scaffold prior to statistical analysis.

#### 2.3.3 DAPI/Phalloidin Staining

To further analyze cell morphology and cytoskeletal organization of SaOS‐2 on the scaffolds, 4′,6‐diamidino‐2‐phenylindole (DAPI) and Phalloidin staining were performed. The cell‐seeded scaffolds were first fixed with 4% paraformaldehyde for 45 min at room temperature. Following fixation, the samples were permeabilized with 0.1% Triton X‐100 for 5 min and then blocked with 1% bovine serum albumin (BSA; Sigma‐Aldrich, St. Louis, MO, USA) for 60 min. For F‐actin staining, the scaffolds were incubated with Phalloidin‐488 (Invitrogen, Waltham, MA, USA) for 60 min in the dark at room temperature. Nuclear staining was performed using 4',6‐diamidino‐2‐phenylindole (DAPI; Sigma‐Aldrich, St. Louis, MO, USA) for 5–10 min at room temperature. High‐resolution fluorescence images were captured at Keyence BZ 9000 (Osaka, Japan).

### 2.4 Mineralization Testing

#### 2.4.1 Calcium Assay in Medium/Scaffold

To quantify calcium deposition on the scaffolds, a calcium assay was employed. After culturing SaOS‐2 cells on MEW‐printed PCL scaffolds for 25 days, the cell‐seeded scaffolds were carefully washed with phosphate‐buffered saline (PBS; Gibco, Thermo Fisher Scientific, Waltham, MA, USA). For both the washed cell‐cultured scaffold samples and CPC‐coated scaffold samples, 100 µL of 0.24 N HCl was added and incubated on a shaker for 24 h with parafilm sealing. Additionally, cell culture medium samples were collected on days 3, 7, 11, 14, 17, 21 and 25 for calcium quantification within the media. Medium samples were collected immediately before medium exchange, after gentle mixing of the well content to ensure homogeneous sampling.

Calcium content analysis was performed using the colorimetric method Fluitest CA CPC (Analyticon, Germany) according to the manufacturer's instructions. 10 µL of supernatant were mixed with the buffer and the chromogen supplied in the kit, incubated on a shaker for 15 min, and then the absorbance was measured at 570 nm using a microplate reader (Infinite M200Pro, Tecan, Switzerland). Calcium content was quantified using a standard curve.

#### 2.4.2 ARS Staining

ARS staining was used to assess the mineralization of both SaOS‐2 cell‐seeded scaffolds and CPC‐coated scaffolds. On day 25 of culture, the scaffolds were fixed in 10% formaldehyde at room temperature for 10 min. Subsequently, the scaffolds were incubated with a 0.1% Alizarin Red S solution (pH 4.2) for 10 min and washed by rinsing with distilled water until the remaining staining solution was removed. Stained scaffolds were imaged with microscopy (Keyence BZ 9000, Osaka, Japan). ARS staining was used here primarily as a qualitative readout of mineral distribution; quantitative mineral content was assessed by calcium extraction (Section 2.4.1)

### 2.5 Mechanical Testing

Tensile tests were conducted using a universal testing machine (Z010 equipped with a 100 N load cell, ZwickRoell, Germany). Each sample was mounted between two grips, and testing was conducted at a constant strain rate of 5 mm/min until failure at room temperature by testXpert II (ZwickRoell, Germany). To mimic the anisotropic mechanical forces experienced by bone tissues, uniaxial tensile tests were performed in both horizontal (parallel to the *x*‐axis (0°)) and vertical (perpendicular to the *x*‐axis (90°)) directions. Stress‐strain curves and force‐displacement data were recorded, and mechanical properties such as ultimate tensile strength and elongation at break were determined.

### 2.6 Patient‐Derived Osteosarcoma on Coated Scaffolds

Osteosarcoma tissue was obtained from tumor resection following informed consent from the patient, approved by the Ethics Committee (approval ID: BO‐EK‐57022020). Tumor tissue was processed by cutting it into small pieces and digesting it using the Human Tumor Dissociation Kit (Miltenyi Biotec, Germany) for 25 min at 37°C and 800 rpm shaking. The cell suspension was centrifuged at 300 g for 5 min. After aspirating the supernatant, the cell pellet was resuspended in fresh medium and centrifuged again. The tumor cells were counted. Coated PCL scaffolds were placed into ultra‐low attachment 24‐well plates and 5 × 10^5^ osteosarcoma cells were added in medium containing epidermal growth factor (EGF; 20 ng/ml), fibroblast growth factor (FGF; 20 ng/ml), and platelet‐derived growth factor A (PDG‐AA; 10 ng/ml). After 7 days, cell culture was stopped by washing the cells with PBS. After fixation with 4% paraformaldehyde, the cell membrane was permeabilized using 0.1% Triton X100 in PBS for 20 min at room temperature, followed by incubation with 1% BSA/0.05% Tween20 in PBS for 10 min at room temperature. F‐actin in the cytoskeleton was stained by phalloidin‐488, and nucleic acids were stained by DAPI. Fluorescence images were captured using the Keyence BZ 9000 (Osaka, Japan).

### 2.7 Statistical Analysis

All statistical analyses were performed using GraphPad Prism (version 10.1.2). All experiments were performed in triplicate. Data are presented as mean ± standard deviation (SD). The coefficient of variation (CV) was used to assess the relative variability of the data. T‐tests were used for two‐group comparisons, and one‐way or two‐way ANOVA followed by post‐hoc tests (e.g., Tukey's, if not stated otherwise) were applied for multi‐group comparisons. Discrepancies in the results when using other post‐hoc tests are mentioned where applicable. A p‐value of less than 0.05 was considered statistically significant. Specifically, *p* < 0.05 is denoted by ^*^, *p* < 0.01 by ^**^, *p* < 0.001 by ^***^, and *p* < 0.0001 by ^****^.

## 3 Results

### 3.1 Characterization of MEW‐printed PCL Scaffolds

**FIGURE 1:**
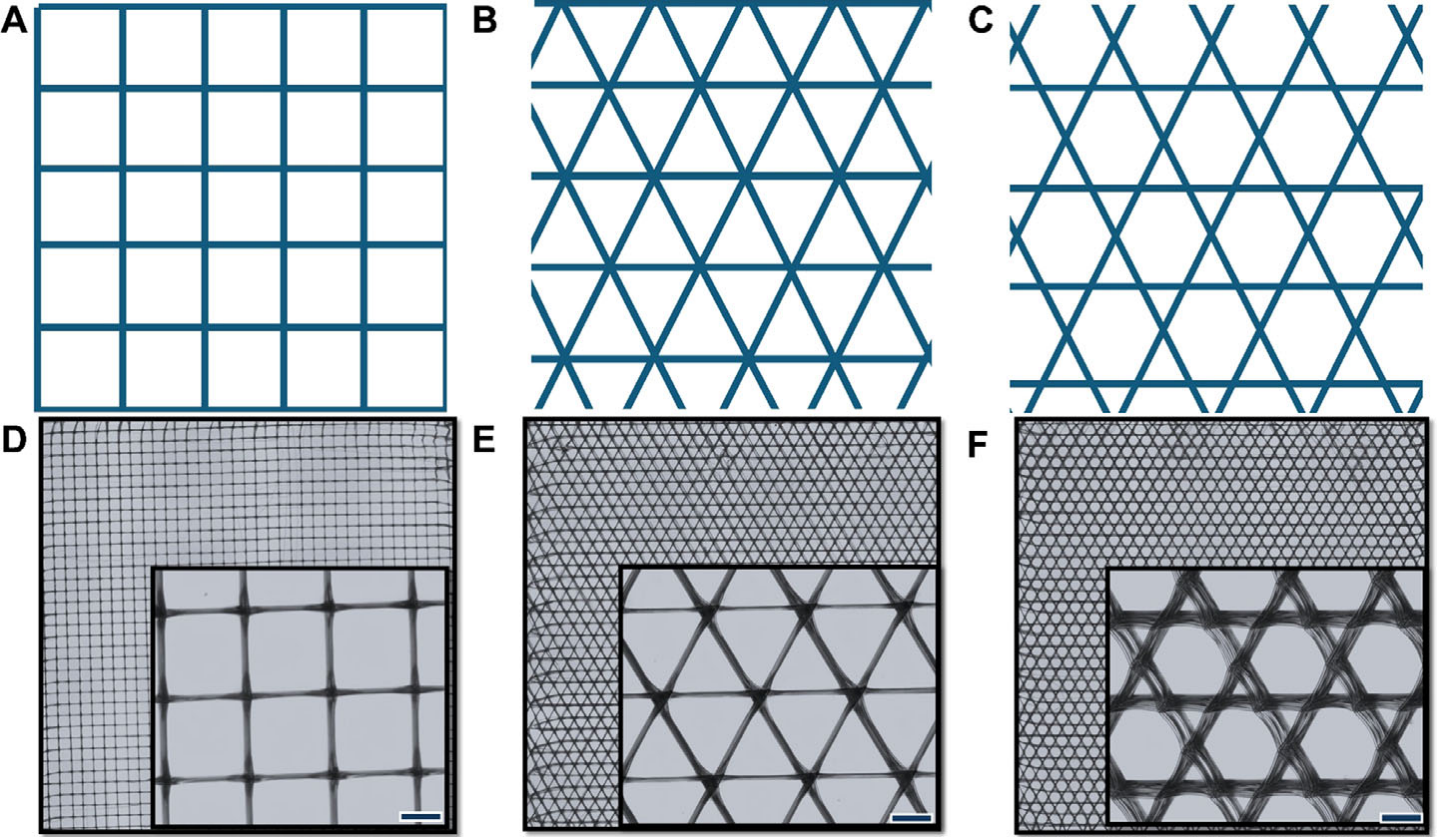
MEW‐fabricated scaffold structures. (A‐C) Schematic designs of rectangular, triangular, and hexagonal structures. (D‐F) Optical microscope images of the scaffolds. Pore size refers to the primary pore opening defined by the center‐to‐center fiber spacing (400 µm), and the fiber diameter is ∼15 µm. The hexagonal structure additionally includes secondary triangular openings of ∼200 µm within the unit. Scale bar: 200 µm.

Using MEW, we fabricated three scaffold architectures (R/T/H) with a nominal center‐to‐center fiber spacing of ∼400 µm and a fiber diameter of ∼15 µm (Figure 1; Table 1). The hexagonal design additionally contains secondary triangular openings (∼200 µm) within each unit cell. For mechanical testing, scaffolds were printed with 40 layers (40L), while cell culture experiments were conducted using scaffolds of varying thicknesses (6L, 24L, and 100L); the scaffold specifications used for each experimental application are summarized in Table 2.

**TABLE 2:** Scaffold specifications for experimental applications.

Application	Layers	Dimensions (L×W)	Note
Live/Dead, DAPI/Phalloidin, ARS staining	6L	7 mm × 7 mm	All three architectures
Calcium quantification	6L, 24L, 100L	7 mm × 7 mm	All three architectures
Mechanical testing	40L	15 mm × 15 mm	All three architectures

Stereo microscopy analysis revealed uniform fiber deposition across all designs. By altering the layer‐to‐layer orientations, we achieved three architectures—rectangular (parallel layers forming square pores), triangular (60° rotations creating triangular pores), and hexagonal (offset 60°/120° layers producing a central hexagon surrounded by triangles). Relevant measurements are summarized in Table 1. In addition to fiber diameter and nominal spacing, projected fiber length density was included as a geometry‐derived parameter to better capture architecture‐dependent differences in fiber content per projected area. While the nominal center‐to‐center spacing was kept comparable across designs, projected fiber length density varied inherently with pore geometry and was highest for the hexagonal architecture.

### 3.2 Characterization of Calcium Phosphate Formation

Calcium phosphate coating of the PCL scaffolds was conducted either by dip‐coating with CPC or by a cell‐derived calcium phosphate deposition using the SaOS‐2 cell line.

#### 3.2.1 Culture Medium Calcium Content Analysis

**FIGURE 2:**
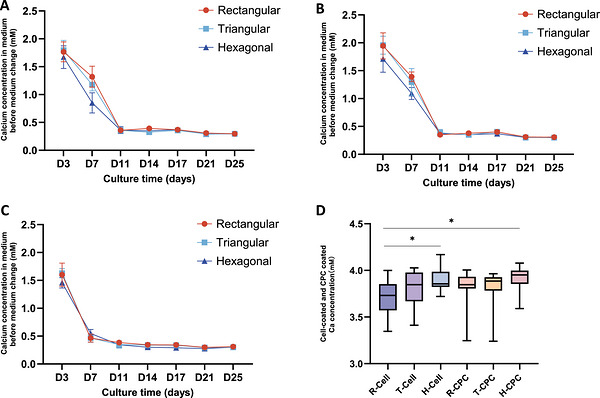
Calcium dynamics in culture medium and calcium deposition on scaffolds. (A–C) Time‐course analysis of calcium ion concentrations in the culture medium for 6‐layer (A), 24‐layer (B), and 100‐layer (C) scaffolds with rectangular, triangular, and hexagonal architectures over 25 days. Time is shown on the *x*‐axis and calcium concentration on the *y*‐axis. The line plots show temporal changes between sampling points rather than continuous real‐time calcium kinetics. (D) Quantitative analysis of calcium content extracted from scaffolds after 25 days. The results compare CaP deposition by SaOS‐2 cells and CPC coating for different geometries (R‐cell: rectangular cell‐cultured, T‐cell: triangular cell‐cultured, H‐cell: hexagonal cell‐cultured, R‐CPC: rectangular CPC‐coated, T‐CPC: triangular CPC‐coated, H‐CPC: hexagonal CPC‐coated). The original day‐by‐day bar graphs are provided in Figure S1.

The calcium ion concentration in the culture medium showed distinct temporal and scaffold‐dependent patterns over the 25‐day observation period (Figure 2A–C; Figure S1). Initial calcium concentrations differed among layer numbers, with 6‐layer scaffolds showing the highest values, followed by 24‐layer scaffolds, whereas 100‐layer scaffolds displayed lower initial concentrations (Table 3). Across all geometries, calcium concentration decreased over time and could be divided into three phases: a rapid reduction phase (Day 3–7), an intermediate phase (Day 7–14), and a steady‐state phase (Day 14–25), during which concentrations stabilized at approximately 0.3–0.5 mmol/L. Overall, higher layer numbers were associated with lower calcium concentrations in the medium due to an increased calcium deposition on the scaffolds, and hexagonal scaffolds tended to exhibit lower medium calcium levels than rectangular scaffolds, particularly in the 24‐ and 100‐layer groups.

**TABLE 3:** Calcium ion concentrations (mmol/L) in culture medium on day 3 for different scaffold geometries and layer numbers (mean ± SD).

Layer Number	Hexagonal	Triangular	Rectangular
6	1.797 ± 0.195	1.942 ± 0.164	1.980 ± 0.387
24	1.774 ± 0.168	1.762 ± 0.334	1.791 ± 0.447
100	1.502 ± 0.089	1.587 ± 0.074	1.459 ± 0.194

Notably, variability was greatest at early time points (Day 3–7) and decreased as values converged toward the steady‐state phase. This early variability likely reflects a combination of (i) a rapid initial uptake/precipitation phase during the onset of osteogenic mineralization, (ii) spatially heterogeneous CaP deposition on scaffolds at early stages, and (iii) the different scaffold designs that affect both CPC deposition and SaOS‐2‐related mineralization. Importantly, the overall trends were consistent with calcium extraction from scaffolds at Day 25 (Figure 2D), supporting scaffold‐dependent mineral accumulation.

#### 3.2.2 Scaffold Calcium Content Analysis

Based on the quantitative analysis after 25 days, the calcium content measurements (Figure 2D) highlighted the combined influence of cellular activity and scaffold geometry on mineralization. In the cell‐cultured scaffolds, the mean calcium contents were 3.913 ± 0.129 mmol/L (hexagonal), 3.818 ± 0.182 mmol/L (triangular), and 3.738 ± 0.194 mmol/L (rectangular) structures, with coefficients of variation (CV) of 3.29%, 4.77%, and 5.19%, respectively. For the CPC‐coated scaffolds, the mean calcium contents were hexagonal (3.933 ± 0.114 mmol/L), triangular (3.808 ± 0.228 mmol/L), and rectangular (3.831 ± 0.219 mmol/L) structures, with CV values of 2.90%, 5.99%, and 5.72%, respectively. Notably, the hexagonal structure consistently exhibited the highest calcium content and the lowest variability in both cell‐cultured and CPC‐coated groups.

#### 3.2.3 Alizarin Red Staining Results

**FIGURE 3:**
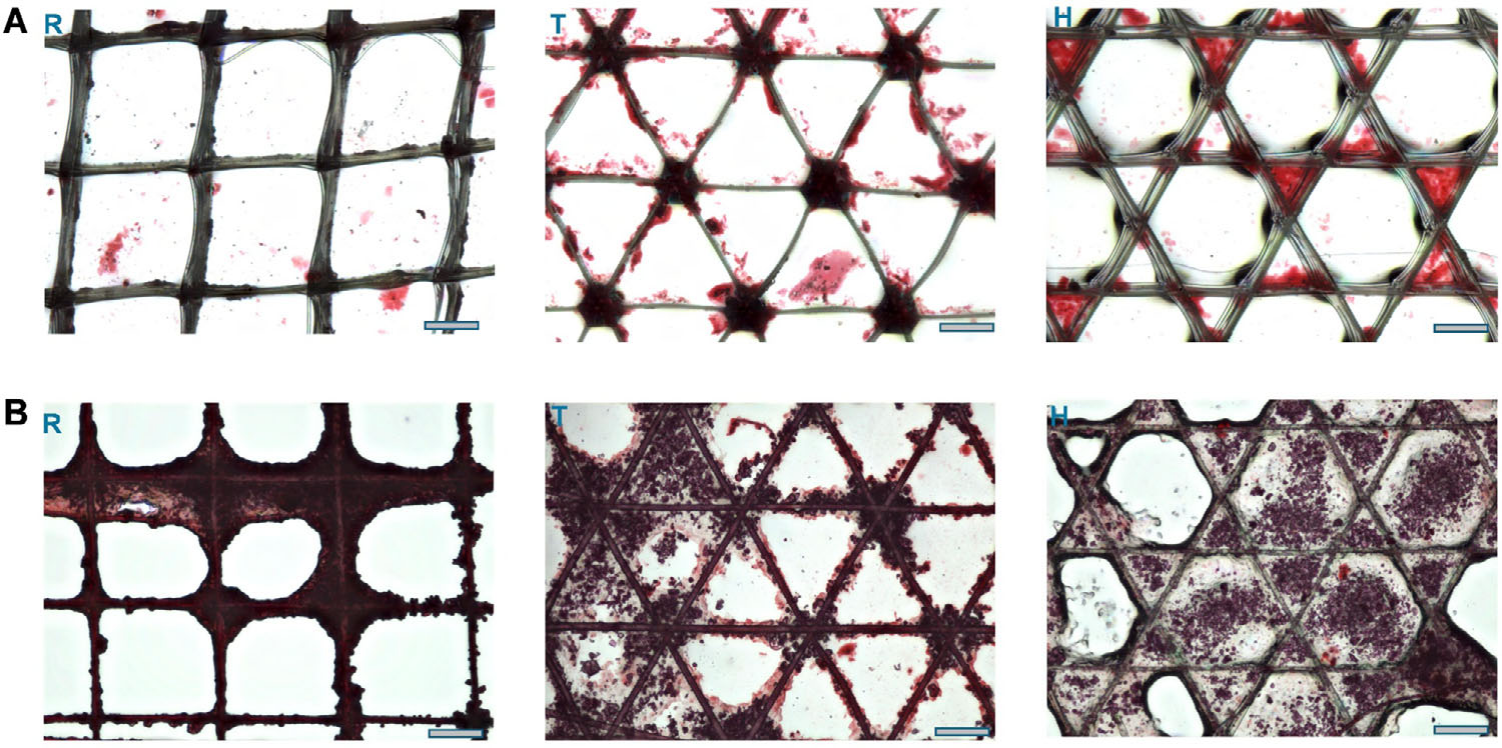
ARS staining of mineralized scaffolds. (A) CPC‐coated scaffolds: ARS highlights the distribution of the cement‐derived CaP phase; deposits were mainly observed at intersections and along pore boundaries. (B) Cell‐mediated mineralization (SaOS‐2, day 25): ARS indicates mineral deposition associated with the cell‐derived matrix; staining was more continuous on fibers and within pores. Scale bar: 200 µm.

ARS staining (Figure 3A,B) showed geometry‐dependent mineral distribution for both mineralization approaches. For CPC‐coated scaffolds (Figure 3A), ARS‐positive regions mainly reflected the spatial distribution of the cement‐derived CaP phase and appeared as localized deposits, predominantly at fiber junctions and along pore boundaries. For cell‐mediated mineralization (Figure 3B), ARS staining was more continuous on the fibers and within pores, indicating mineral deposition associated with the cell‐derived matrix. Across geometries, the hexagonal design showed the most homogeneous staining pattern, whereas the triangular and rectangular designs displayed more localized staining, particularly at intersections.

### 3.3 Mechanical Properties of MEW‐Printed Scaffolds

#### 3.3.1 Overall Tensile Test Comparison

**FIGURE 4:**
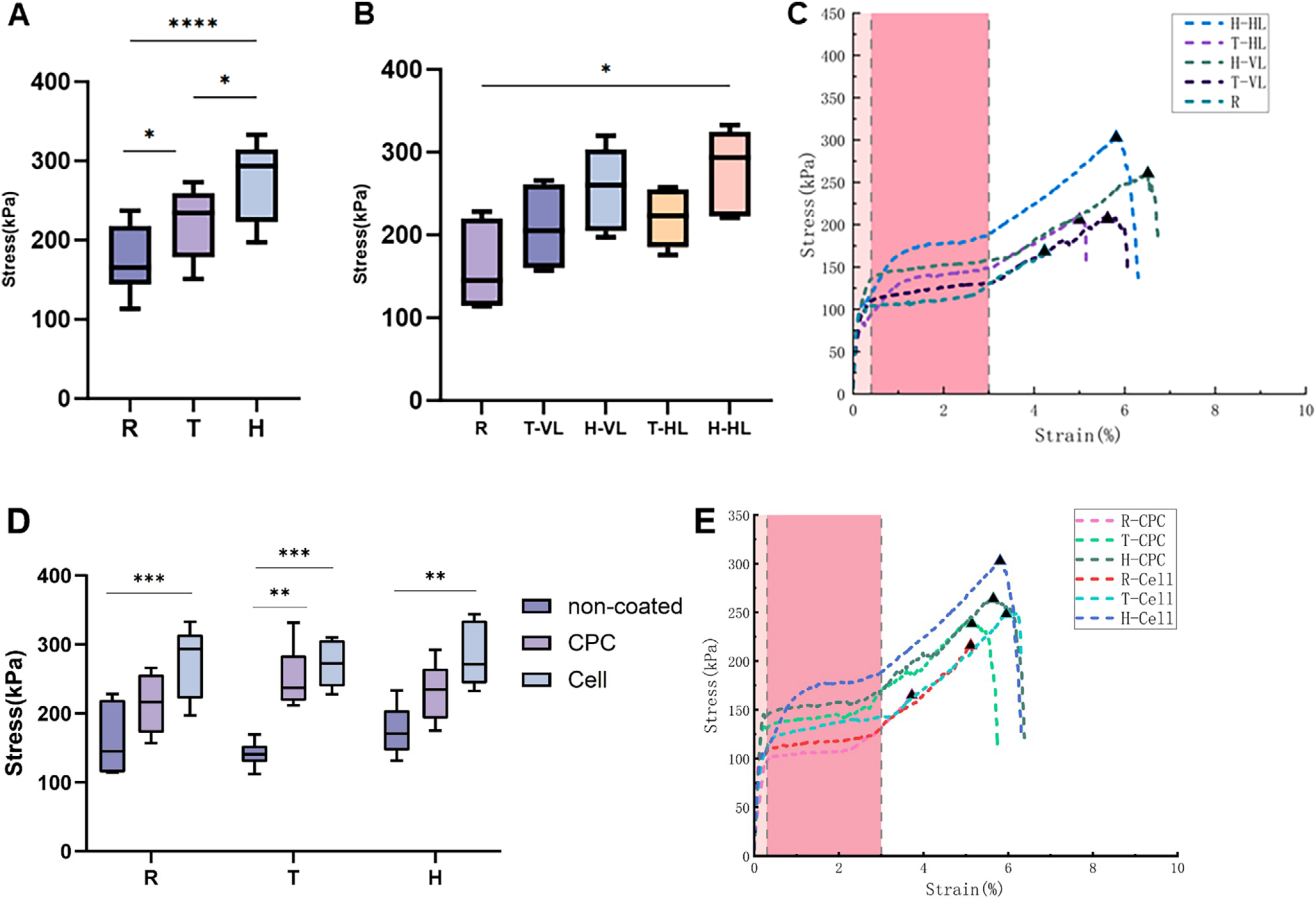
Tensile behavior and deformation regimes of MEW‐printed PCL scaffolds. (A) Overall ultimate tensile strength (UTS) pooled from horizontal and vertical loading. (B) Direction‐dependent UTS (HL: horizontal loading; VL: vertical loading; R: rectangular; T: triangular; H: hexagonal). (C) Representative stress–strain curves for directional loading. Shaded areas indicate the initial toe and quasi‐linear regions; peak markers indicate UTS. (D) Effect of coating/mineralization on UTS (uncoated vs SaOS‐2‐derived CaP matrix (“Cell”) vs CPC). (E) Representative stress–strain curves illustrating coating‐dependent changes; Zone I–II shading and peak markers are defined as in (C), and Zones III–IV follow the same peak‐referenced definition.

Because the three patterns differ in junction density and effective fiber content per unit area, differences in tensile strength should be interpreted in the context of geometry‐dependent load‐sharing pathways. The tensile strength analysis revealed significant differences among the three structural designs in both overall and directional comparisons. Here, overall tensile strength refers to values pooled from horizontal and vertical tests. In terms of overall tensile strength, the hexagonal structure demonstrated the highest performance (269.9 ± 49.61 kPa, n = 12), significantly outperforming the triangular structure (221.4 ± 43.57 kPa, n = 12) and the rectangular structure (174.7 ± 42.63 kPa, n = 12). These results indicate that, among the tested designs, the investigated hexagonal architecture showed the highest overall tensile strength, followed by the triangular and rectangular architectures (Figure 4A).

#### 3.3.2 Directional Comparison

In the directional comparison, the rectangular structure (R, 160.7 ± 49.92 kPa) exhibited significantly lower tensile strength compared to the hexagonal structure under horizontal loading (H‐HL, 281.0 ± 48.43 kPa) (p < 0.01). For both the triangular (T) and hexagonal (H) scaffolds, the tensile strength under horizontal loading (T‐HL and H‐HL) was higher than that under vertical loading (T‐VL and H‐VL), although the differences were not statistically significant (p > 0.05) (see Figure 4B,C). The representative stress–strain curves showed an initial toe region followed by a quasi‐linear region before reaching UTS; the mechanistic interpretation of these deformation regions is discussed below.

#### 3.3.3 Effect of Calcium Phosphate Formation

Based on the data given in Figure 4D,E, scaffolds with different micro‐architectures exhibited significant differences in tensile strength under various coating treatments. For rectangular scaffolds, the cell‐coated group showed the highest tensile strength (175.5 ± 35.73 kPa), which was significantly higher than the uncoated group (160.7 ± 49.92 kPa, p < 0.05) and the CPC‐coated group (141.2 ± 18.94 kPa, p < 0.01). For triangular scaffolds, the CPC‐coated group demonstrated the best performance (251.0 ± 44.34 kPa), significantly higher than the uncoated group (214.7 ± 43.62 kPa, p < 0.05) and the cell‐coated group (231.8 ± 41.92 kPa, p < 0.05). For hexagonal scaffolds, the tensile strength differences among the three groups were relatively small, with the cell‐coated group (282.8 ± 45.62 kPa) slightly higher than the CPC‐coated group (271.6 ± 32.71 kPa) and the uncoated group (269.0 ± 50.41 kPa). Statistical results suggest that coating treatments significantly enhanced the mechanical performance of rectangular and triangular scaffolds, while the hexagonal scaffolds showed no significant differences among the groups.

### 3.4 Cell Behavior and Biological Characterization

**FIGURE 5:**
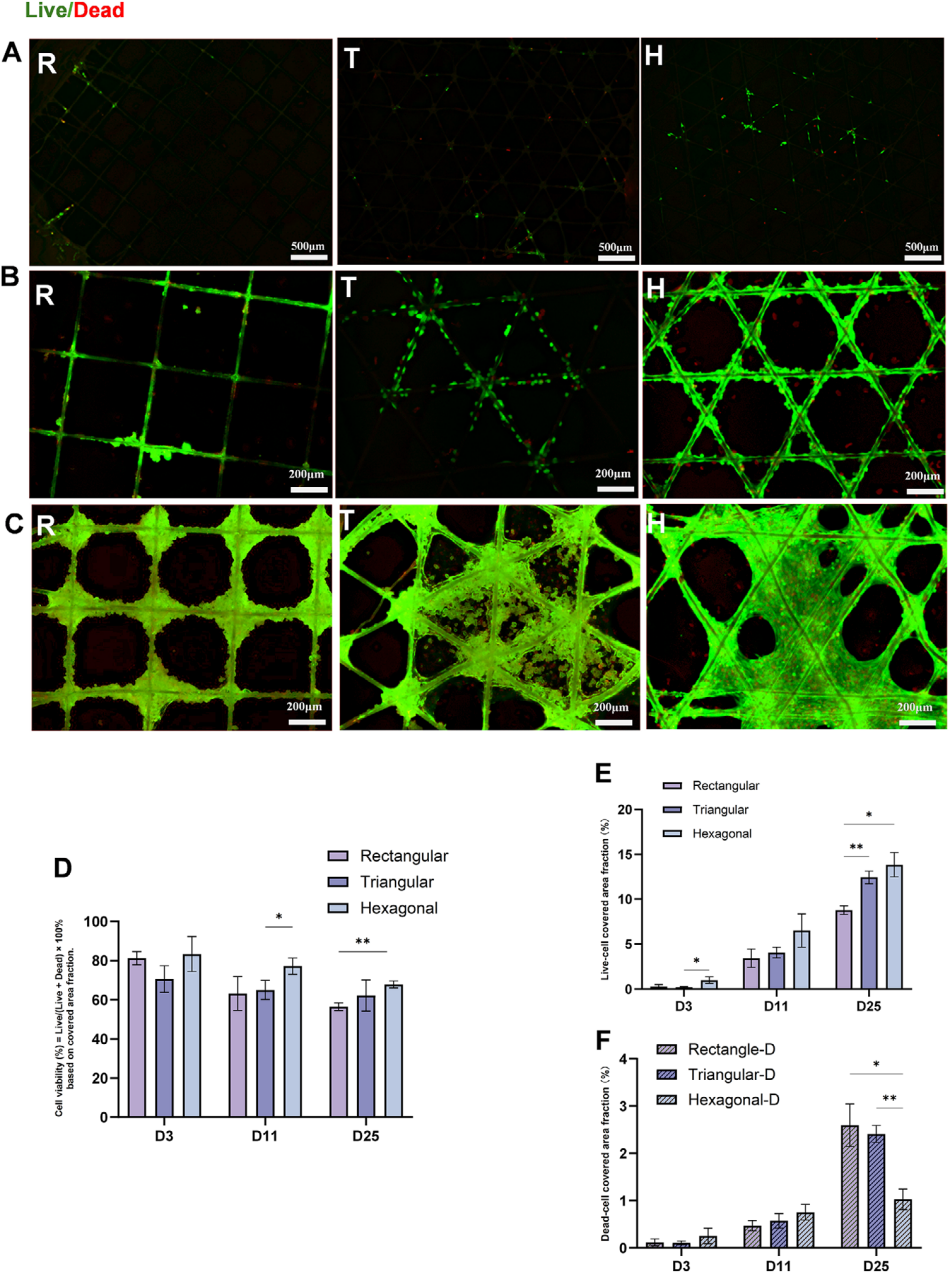
Viability of SaOS‐2 cells cultured on MEW scaffolds with different geometries. (A–C) Representative Live/Dead fluorescence images of rectangular (R), triangular (T), and hexagonal (H) scaffolds at Day 3 (A), Day 11 (B), and Day 25 (C) (left to right). Live cells were stained with calcein‐AM (green) and dead cells with EthD‐1 (red). (D) Viability calculated from covered area fraction as Live/(Live + Dead) × 100%. (E) Live‐cell covered area fraction within the visible scaffold region. (F) Dead‐cell covered area fraction within the visible scaffold region. (n = 3, 3 fields of view per scaffold, mean ± SD, ^*^ *p* < 0.05, ^**^ *p* < 0.01).

Live/Dead staining showed that SaOS‐2 cells remained predominantly viable on all scaffold geometries over 25 days of culture (Figure 5A–C). From Day 3 to Day 25, the extent of green fluorescence increased in all groups, indicating progressive cell coverage of the scaffold surfaces. Quantification based on area fractions confirmed a time‐dependent increase in the live‐cell covered area fraction, whereas the dead‐cell area fraction remained low throughout the culture period (Figure 5D–F). This area‐based analysis directly supports the image‐based observation that cell coverage increased over time and was highest on the investigated hexagonal architecture at later culture time points.

At later time points, qualitative inspection suggested geometry‐dependent differences in spatial distribution. On rectangular scaffolds, cells were mainly detected along individual fiber surfaces, with comparatively limited signal spanning across pore openings. In contrast, triangular and hexagonal scaffolds exhibited more continuous green signal across the visible scaffold region and more frequent pore‐bridging features by Day 25 (Figure 5C).

**FIGURE 6:**
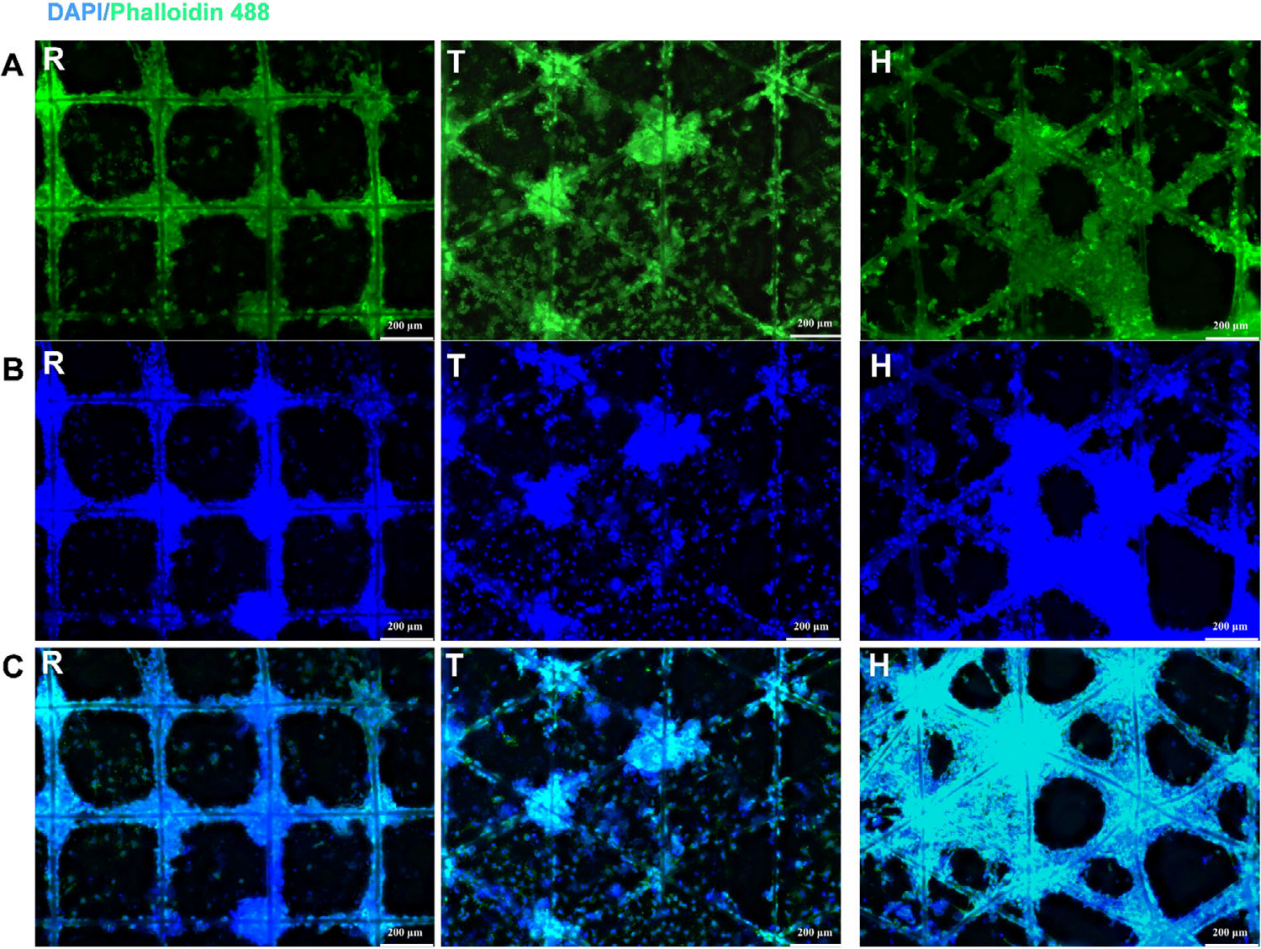
Cytoskeletal organization and nuclear distribution of SaOS‐2 cells on MEW scaffolds. Representative fluorescence images at Day 25 for rectangular (R), triangular (T), and hexagonal (H) scaffolds (left to right). (A) Phalloidin 488 (F‐actin, green), (B) DAPI (nuclei, blue), and (C) merged images. Scale bar: 200 µm.

DAPI/Phalloidin staining at Day 25 further supported these observations by showing widespread nuclear presence and pronounced cytoskeletal organization across all geometries (Figure 6). F‐actin staining indicated extensive cell spreading along fibers, and merged images showed cells bridging between adjacent fibers in triangular and hexagonal scaffolds, consistent with the distribution patterns observed in the Live/Dead images.

### 3.5 Scaffolds for Osteosarcoma Culture

Patient‐derived osteosarcoma cells were seeded on PCL scaffolds with rectangular, triangular, or hexagonal patterns that were coated either with a SaOS‐2‐derived CaP matrix or with CPC. Cell culture was stopped on day 7, cells were fixed by formaldehyde and stained with DAPI staining and F‐actin staining.

**FIGURE 7:**
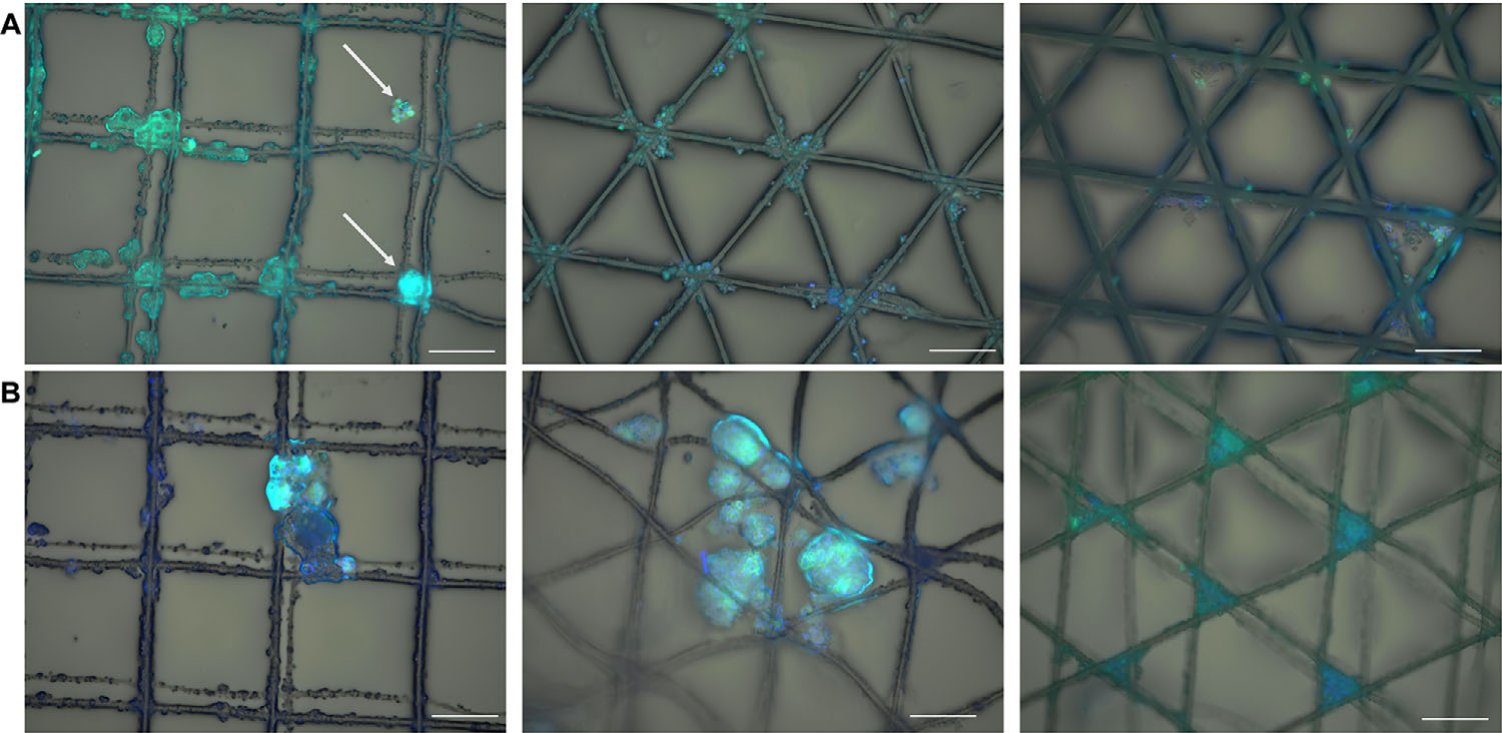
Patient‐derived osteosarcoma cells were cultured for seven days on PCL scaffolds that were coated either with a SaOS‐2‐derived calcium phosphate matrix (A) or with CPC (B). The cytoskeleton was stained with phalloidin‐488 (green), and the nuclei were stained using DAPI (blue). Arrows indicate representative cell clusters/aggregates. Scale bars: 200 µm.

The osteosarcoma cells adhered to the coated PCL fibers with differences in cell number and location. On the triangular and rectangular patterns, osteosarcoma cells settled preferably to the fiber intersections (Figure 7A). On the CPC‐coated scaffolds, the cells formed bigger clusters that adhered to the intersections and the arms of the triangles and rectangles (Figure 7B). On the SaOS‐2‐coated hexagonal structures, the osteosarcoma cells adhered in smaller clusters to the fibers with no preference to intersections or free fibers (Figure 7A, right). When the fibers were coated with CPC, cells were preferentially found in the tips of the hexagons (Figure 7B, right).

## 4 Discussion

Osteosarcoma is the most common primary bone cancer in children and adolescents [39]. Despite progress in surgery, chemotherapy, and radiotherapy, patient prognosis remains strongly associated with the response to polychemotherapy [40], which is considered a major predictor of overall survival [41].

In this study, we successfully fabricated PCL scaffolds with three different geometric shapes (rectangular, triangular, and hexagonal) using melt electrowriting. While previous studies have demonstrated the mechanical and biological advantages of these architectures, our results further confirm their suitability for bone tissue engineering [42, 43, 44]. The chosen pore size of 400 µm falls within the optimal range of 300–500 µm reported for promoting osteoblast activity and nutrient exchange [45], supporting the potential of these designs for bone regeneration applications.

### 4.1 Scaffold Geometry and Coating Technique Effects on Mineralization

Our findings show that both scaffold geometry and layer number influenced mineralization efficiency. Consistent with previous reports, increasing the number of scaffold layers from 6 to 100 enhanced mineralization capacity, likely due to the increased surface area available for cell attachment and calcium phosphate deposition [46, 47]. This was reflected by greater calcium deposition on thicker scaffolds and lower calcium ion concentrations in the culture medium. The larger spread in medium calcium concentration at early time points likely reflects the dynamic nature of mineralization, which often proceeds through an initial burst phase followed by a transition toward equilibrium. In osteogenic media, transient micro‐precipitation may also contribute to variability; therefore, medium calcium trends should be interpreted together with the steady‐state plateau and the scaffold calcium extraction data (Figure 2D), which confirm the scaffold‐dependent deposition trend.

ARS staining should be interpreted differently for the two mineralization approaches. For CPC‐coated scaffolds, ARS primarily reflects the presence and distribution of the cement‐derived CaP phase, whereas for cell‐mediated mineralization it indicates mineral deposited in association with the cell‐derived matrix [48]. Consistent with the calcium assay, ARS staining showed architecture‐dependent mineral distribution. The investigated hexagonal architecture showed the most homogeneous staining pattern, whereas triangular and rectangular scaffolds showed more localized deposition, particularly at intersections. These observations suggest that layer number and architecture‐dependent fiber organization influence mineral accumulation, likely through differences in available surface area, projected fiber length density, and junction distribution [49, 50].

### 4.2 Geometric Design and Coating Effects on Scaffold Mechanics

The mechanical properties of scaffolds are strongly influenced by their geometric shapes. Tensile strength analysis revealed that the hexagonal scaffold had the highest tensile strength, significantly exceeding that of the triangular and rectangular scaffolds. This superior performance may be related to the combination of its complex pore organization, higher projected fiber length density, and geometry‐dependent junction and load‐sharing characteristics, which together support more effective stress distribution and structural stability. In directional force analysis, the hexagonal scaffold exhibited significantly higher tensile strength under horizontal loading than the rectangular scaffold. While its strength exceeded that of the triangular scaffold across directions, the differences were not statistically significant. These findings are consistent with the broader MEW literature showing that fiber orientation, pore architecture, fiber spacing, and fiber density can strongly influence scaffold stiffness, load‐bearing behavior, and anisotropic mechanical response [51]. These findings are also relevant for bone‐mimetic scaffold design, where multidirectional mechanical behavior is important because bone tissue experiences complex loading conditions [52, 53].

Importantly, the stress–strain curves exhibited a conserved principal shape across geometries (Figure 4C,E), which can be interpreted by considering deformation “zones” during tensile loading. The initial toe region (Zone I) is consistent with fiber straightening and junction rotation toward the loading axis, followed by a quasi‐linear regime (Zone II) dominated by stretching of load‐bearing fiber segments. At higher strains, deviation from linearity (Zone III, defined relative to the peak marker) suggests progressive junction rotation/sliding and local structural rearrangement as load paths redistribute within the micro‐architecture. After the peak stress (Zone IV), damage accumulation and progressive failure lead to the descending response. This zone‐based interpretation provides a mechanistic link between the measured tensile response and how MEW micro‐fiber networks deform under tension, while the absolute stress level and the extent of each zone remain geometry‐dependent due to differences in projected fiber length density, junction frequency, and load‐sharing paths.

Coating treatments also influenced tensile behavior in an architecture‐dependent manner. For rectangular scaffolds, the cell‐coated group showed the highest tensile strength, whereas CPC coating was most effective in triangular scaffolds [54]. In contrast, the investigated hexagonal architecture showed only minor differences among coating groups, suggesting that its baseline architecture already provided efficient load‐sharing. This may be related to the local stress distribution enabled by the smaller triangular openings within the investigated hexagonal architecture [55], whereas rectangular scaffolds may be more affected by stress concentration and fewer load‐sharing pathways [56, 57, 58]. These results indicate that scaffold architecture and mineral phase should be considered together when designing mechanically robust bone‐mimetic platforms.

### 4.3 SaOS‐2 Cell Behavior on PCL Scaffolds

The Live/Dead assay demonstrated that SaOS‐2 cells remained predominantly viable on all three architectures over 25 days, with increasing live area fraction over time and persistently low dead area fraction (Figure 5). This indicates that MEW‐printed PCL scaffolds provide a permissive substrate for long‐term culture under the conditions tested. At the same time, qualitative inspection suggested geometry‐dependent differences in spatial distribution at later time points (Figure 5C and Figure 6), highlighting that micro‐architecture can modulate how cells populate and bridge pores even when overall viability is high.

Architecture influenced the spatial distribution of SaOS‐2 cells. Rectangular scaffolds showed cell coverage mainly along individual fibers and limited pore bridging, likely because larger uninterrupted pore regions and fewer junction‐rich anchoring sites limited cell spanning across pores [59].

In the triangular scaffolds, Live/Dead images showed predominantly viable cells throughout culture, with increasing coverage over time (Figure 5). Compared with rectangular scaffolds, triangular designs appeared to support more continuous coverage at later time points, and pore‐spanning features were more frequently observed by Day 25 (Figure 5C). These observations suggest that the triangular architecture provides junction‐rich anchoring sites that can facilitate cell spreading and bridging across openings, while overall viability remained high across all geometries.

Among the tested designs, the investigated hexagonal architecture showed the highest live‐cell covered area fraction and the most continuous cell distribution at later time points. However, this effect should not be interpreted as a consequence of hexagonal pore shape alone. The hexagonal design used in this study contains primary hexagonal openings together with secondary triangular openings of approximately 200 µm, resulting in smaller local spanning distances, increased junction availability, and higher projected fiber length density. These combined architectural features likely facilitated cell attachment, pore bridging, and more continuous cell coverage [59]. In contrast, rectangular scaffolds provided fewer junction‐rich anchoring sites and larger uninterrupted pore regions, which may have limited cell bridging. Therefore, the observed differences in SaOS‐2 distribution are best interpreted as architecture‐level effects arising from the combined influence of pore organization, local spacing, fiber density, and junction distribution.

### 4.4 Behavior of Patient‐derived Osteosarcoma Cells Depends on Structural Pattern

Patient‐derived osteosarcoma cells were included as a translational proof‐of‐concept readout to evaluate whether mineralized MEW scaffolds support primary tumor cell attachment and architecture‐dependent distribution. These experiments differed from the SaOS‐2 experiments in several important aspects. SaOS‐2 cells were used as a standardized osteogenic osteosarcoma cell line for scaffold screening, long‐term culture, and cell‐mediated mineralization over 25 days. In contrast, patient‐derived osteosarcoma cells were cultured for 7 days on pre‐mineralized scaffolds. Therefore, the two systems cannot be directly compared in terms of proliferation kinetics.

The present observations suggest that scaffold architecture, pore organization, and coating material influenced patient‐derived osteosarcoma cell attachment and distribution. Smaller or more junction‐rich pore regions may provide more available attachment sites and support local cell aggregation. Although fluid flow in smaller pores may affect shear stress, the observed distribution patterns are more likely related to differences in available surface area and attachment sites [60, 61]. Such local structural features may also facilitate cell aggregate or spheroid‐like formation [62], which is relevant because 3D spheroid culture can better mimic selected physiological features than conventional 2D culture [63].

Coating material further affected cell distribution. CPC coatings promoted larger cell clusters at scaffold intersections and vertices, particularly in triangular and rectangular architectures. In contrast, SaOS‐2‐derived CaP coatings tended to support a more uniform distribution along the fibers in the investigated hexagonal architecture. These findings highlight that scaffold geometry and coating chemistry jointly influence patient‐derived osteosarcoma cell organization [64].

In addition, the use of 3D scaffolds demonstrates significant advantages over traditional 2D culture systems. These scaffolds can more accurately simulate cell‐cell and cell‐matrix interactions, thereby facilitating the study of key tumor behaviors such as migration, invasion, and drug resistance [65, 66]. To further optimize scaffold performance, future research should explore smaller pore sizes, increase fiber intersection density, and develop composite coatings that combine the advantages of CPC and CaP.

### 4.5 Limitations and Future Work

Several limitations should be acknowledged. First, scaffold thickness was reported as a nominal estimate based on layer number and fiber diameter, rather than being directly measured. Since junction build‐up and architecture‐dependent stacking may affect local scaffold height, future studies should include cross‐sectional microscopy or profilometry.

Second, the mechanical analysis focused on uniaxial tensile testing and tensile anisotropy. Compression or bending tests would provide useful complementary information, but reliable testing of thin, highly open MEW scaffolds requires dedicated fixtures, controlled contact conditions, and accurate thickness measurements. These analyses will therefore be addressed in future work.

Third, this study compares complete scaffold architectures rather than isolated geometric parameters. The investigated hexagonal architecture includes secondary triangular openings, smaller local spacing, higher projected fiber length density, and increased junction availability. Thus, the observed effects cannot be attributed to hexagonal pore shape alone. Future studies should use pore‐spacing‐ and fiber‐density‐matched designs to separate the effects of pore shape, spacing, and junction density. Honeycomb‐like MEW architectures without secondary triangular openings would be useful future controls to isolate the effect of pore shape. However, such patterns may require heterogeneous fiber or strand layer organization during MEW printing, introducing additional fabrication constraints. This will therefore be addressed in future work.

Finally, the patient‐derived osteosarcoma experiments should be considered proof‐of‐concept. Additional patient samples, longer culture periods, and molecular characterization are needed to validate the biological relevance of the observed architecture‐ and coating‐dependent cell distributions.

## 5 Conclusion

This study examined how scaffold architecture, layer number, and mineralization strategy influence the mechanical performance, mineral deposition, and osteosarcoma cell behavior of MEW‐printed PCL scaffolds. Among the tested designs, the investigated hexagonal architecture showed the most favorable overall performance, including high tensile strength, homogeneous calcium deposition, and continuous cell coverage. However, these findings should be interpreted as architecture‐level effects because the hexagonal design also included secondary triangular openings and higher projected fiber length density. Coating effects were also architecture‐dependent, indicating that scaffold geometry and mineral phase should be considered together when designing bone‐mimetic in vitro platforms. Overall, the presented platform provides a promising basis for more physiologically relevant osteosarcoma models using mineralized MEW scaffolds and patient‐derived osteosarcoma cells.

## Funding

This research received no external funding.

## Conflicts of Interest

The authors declare no conflicts of interest.

## Supporting information




**Supporting File**: mabi70224‐sup‐0001‐SuppMat‐FigureS1.docx.

## Data Availability

The data that support the findings of this study are available from the corresponding author upon reasonable request.
