# Supplementary material for: Mimicking the Osteosarcoma Bone Microenvironment Using MEW‐Printed PCL Scaffolds
Source: Macromol Biosci. 2026 Jul 18;26(7):e70224. doi: 10.1002/mabi.70224 (PMC13380633; doi:10.1002/mabi.70224)
Supplement: Supplementary file 1 — Supporting File: mabi70224‐sup‐0001‐SuppMat‐FigureS1.docx. [file MABI-26-e70224-s001.docx]

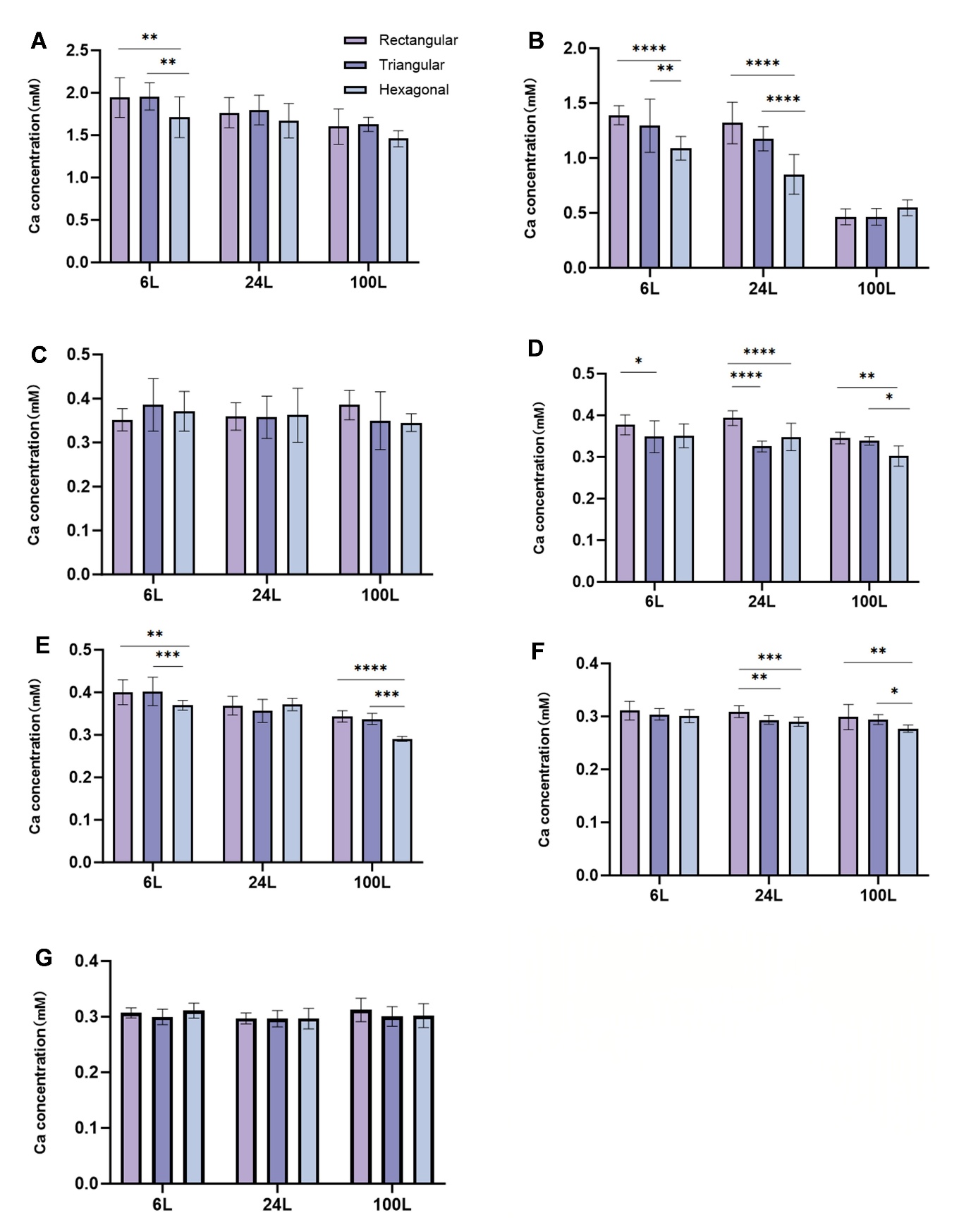
Figure S1. (A-G) Calcium ion concentrations in the culture medium for scaffolds with different geometries (hexagonal, triangular, and rectangular) and layer numbers (6L, 24L, and 100L) over 25 days. The data show temporal and scaffold-dependent variations, with specific trends highlighted at day 3 (A), day 7 (B), day 11 (C), day 14 (D), day 17 (E), day 21 (F), and day 25 (G). (H) Quantitative analysis of calcium content in scaffolds after 25 days. The results compare the CaP deposition by SaOS-2 cells and CPC-coated scaffolds for different geometries (R-cell: rectangular cell-cultured, T-cell: triangular cell-cultured, H-cell: hexagonal cell-cultured, R-CPC: rectangular CPC-coated, T-CPC: triangular CPC-coated, H-CPC: hexagonal CPC-coated).
